# The Adult Separation Anxiety Questionnaire (ASA-27): reliability of the German translation, factor structure, and concurrent validity with anxiety sensitivity and agoraphobic cognition

**DOI:** 10.1007/s00115-025-01806-w

**Published:** 2025-03-17

**Authors:** Patrik D. Seuling, Michael G. Gottschalk, Melanie Vietz, Ulrike Lueken, Tina B. Lonsdorf, Udo Dannlowski, Paul Pauli, Jürgen Deckert, Stefano Pini, Vijaya Manicavasagar, Katharina Domschke, Miriam A. Schiele

**Affiliations:** 1 Department of Psychiatry and Psychotherapy, Medical Center - University of Freiburg, Faculty of Medicine, University of Freiburg, Hauptstraße 5, 79104 Freiburg, Germany; 2 Research Clinic and Outpatient Department, Max Planck Institute of Psychiatry, Munich, Germany; 3 Department of Psychiatry, Psychosomatics and Psychotherapy, University of Würzburg, Würzburg, Germany; 4 Institute of Psychology, Humboldt-Universität zu Berlin, Berlin, Germany; 5 German Center for Mental Health (DZPG), partner site Berlin/Potsdam, Berlin, Germany; 6 Institute of Psychology, Biological Psychology and Cognitive Neuroscience, University of Bielefeld, Bielefeld, Germany; 7 Institute for Systems Neuroscience, University Medical Center Hamburg-Eppendorf, Hamburg, Germany; 8 Institute for Translational Psychiatry, University of Münster, Münster, Germany; 9 Department of Psychology I—Biological Psychology, Clinical Psychology and Psychotherapy, University of Würzburg, Würzburg, Germany; 10 Department of Psychiatry, University of Pisa, Pisa, Italy; 11 Discipline of Psychiatry and Mental Health, School of Clinical Medicine, University of New South Wales, Sydney, Australia

**Keywords:** Anxiety Disorders, SEPAD, Psychometrics, Diagnostics, Separation Phobia, Trennungsangst, Trennungsangststörung, Psychometrie, Diagnostik, Angst

## Abstract

**Background:**

Separation anxiety disorder (SEPAD) is characterized by pronounced fear or anxiety concerning separation from attachment figures. Despite its high lifetime prevalence, adult SEPAD often remains undetected due to a lack of diagnostic tools in multiple languages. The Adult Separation Anxiety Questionnaire (ASA-27) is a key instrument for assessing symptoms of SEPAD in adults. However, no validated German version is available.

**Objectives:**

This study addressed the translation and validation of the ASA-27 in a German-speaking population to introduce the first German questionnaire assessing SEPAD.

**Materials and methods:**

A consecutive forward and backward translation was conducted. Reliability and validity of the German ASA-27 against several established anxiety-related psychometric scores were assessed in a large sample of 1520 healthy participants.

**Results:**

Results revealed robust internal consistency (Cronbach’s α = 0.87) and a factor structure explaining 49.7% of variations in answers. Concurrent validity was confirmed through significant correlations with established anxiety measures. Younger age and female sex were positively correlated with ASA-27 scores.

**Conclusion:**

The German ASA-27 constitutes a promising diagnostic tool for adult SEPAD with sound psychometric properties and a coherent factor structure, offering a structured and reliable assessment of SEPAD and its dimensional evaluation in German-speaking populations.

**Supplementary Information:**

The online version of this article (10.1007/s00115-025-01806-w) contains supplementary material, which is available to authorized users.

## Abstract (de)

**Hintergrund:**

Die Trennungsangststörung (SEPAD) zeichnet sich durch ausgeprägte Angst oder Furcht vor der Trennung von Bezugspersonen aus. Trotz der hohen Lebenszeitprävalenz bleibt SEPAD oft unerkannt, da diagnostische Werkzeuge in vielen Sprachen nicht verfügbar sind. Der Adult Separation Anxiety Questionnaire (ASA-27) ist ein Schlüsselinstrument zum Erfassen von SEPAD-Symptomen bei Erwachsenen, jedoch ist derzeit keine validierte deutsche Version verfügbar.

**Ziel der Arbeit:**

Diese Studie beschreibt die Übersetzung und Validierung des ASA-27 in einer deutschsprachigen Population.

**Material und Methoden:**

Nach einer konsekutiven Vorwärts-Rückwärts-Übersetzung wurden Reliabilität und Validität des deutschen ASA-27 anhand etablierter Angstskalen in einer Stichprobe von 1520 gesunden Probanden untersucht.

**Ergebnisse:**

Es ergab sich eine robuste interne Konsistenz (Cronbachs α = 0,87) und eine Faktorenstruktur, die 49,7 % der Antwortvariation erklärte. Die konkurrente Validität wurde durch signifikante Korrelationen mit etablierten Angstmaßen abgesichert. Jüngeres Alter und weibliches Geschlecht gingen mit höheren ASA-27-Scores einher.

**Diskussion:**

Der deutsche ASA-27 stellt ein geeignetes diagnostisches Werkzeug für SEPAD bei Erwachsenen dar. Er zeigt gute psychometrische Werte sowie eine kohärente Faktorenstruktur und stellt ein strukturiertes und reliables Instrument zur Erfassung von SEPAD in deutschsprachigen Populationen dar.

## Brief introduction to the subject

Since the* Diagnostic and Statistical Manual of Mental Disorders, Fifth Edition* (DSM-5) and *International Classification of Diseases, 11th Revision* (ICD-11) have removed the age-of-onset stipulation of age, separation anxiety disorder (SEPAD) can be diagnosed in adults. However, to date, no questionnaires in German are available for adult SEPAD. The Adult Separation Anxiety Questionnaire (ASA-27) is the leading questionnaire for assessing SEPAD symptoms in adults. We translated the ASA-27 into German and validated it in a sample of 1520 adults. The German ASA-27 has good psychometric properties and is a promising tool for assessing SEPAD in adult German-speaking populations.

## Introduction and background

SEPAD, previously classified as a “disorder usually diagnosed in infancy, childhood, and adolescence” with an onset before the age of 18 years, has been reclassified as an “anxiety disorder” in the DSM-5—with the ICD-11 following suit—after evidence from epidemiological studies reporting 43–78% of adults with the first onset of SEPAD over the age of 18 years [28, 30]. Symptoms of SEPAD in adults are similar to those in children and can include the following [2, 23]:

- Pronounced anxiety regarding separation from attachment figures or places of personal significance
- Pathological worry about harm befalling close attachments
- Frantic efforts to keep in touch with close attachments
- Nightmares or somatic symptoms associated with separation situations
- Refusal to stay at home alone or to leave the house alone, in an effort to avoid separation from attachment figures

SEPAD is characterized by high comorbidity rates with other mental disorders [2, 4, 11, 22], which can result in a challenging differentiation from other mental disorders. Additionally, comorbid SEPAD can complicate treatment of other mental disorders, resulting in reduced therapy response (cf. [1, 10, 11]). Yet, SEPAD often remains undetected and untreated in adults (cf. [11, 22]), possibly due to the limited availability of validated, multilingual instruments to assess symptoms of SEPAD in adult populations.

The Adult Separation Anxiety Questionnaire (ASA-27; [12]) is the first and most widely used psychometrically validated instrument to evaluate SEPAD in adults. It consists of 27 items rated on a four-point scale (*very often, quite often, occasionally*, and *never*), assessing separation anxiety both dimensionally and categorically, with a recommended cut-off score of 22 (of a total possible score of 81) to indicate a probable diagnosis of SEPAD. This value has been empirically determined as the intersection point of the sensitivity and specificity curves for the original English questionnaire (cf. [12]). The measure has good psychometric properties (Cronbach’s α = 0.95; test–retest reliability: *r* = 0.86 [12]) and has already been validated in Turkish [5], Spanish [18], and Portuguese [19], but so far, not in German. Here, we report on (a) the translation of the ASA-27 into German, (b) its reliability and factor structure, and (c) its concurrent validity against other questionnaires assessing symptoms of anxiety and depression.

## Study design and investigation

### Sample and psychometric characterization

All participants were part of the CRC-TRR58 subproject Z02. For a detailed description of the recruitment process and the selection criteria, see [23]. Participants were recruited from the general population using various methods such as advertising through flyers, online platforms, poster displays, or word-of-mouth referrals. The absence of currently manifest or lifetime mental axis I disorders was established by trained clinical psychologists via the Mini-International Neuropsychiatric Interview (MINI) following DSM-IV criteria [29]. In brief, inclusion criteria were: Caucasian family background (based on self-report, for up to three past generations), right-handedness, and fluency in German. Exclusion criteria were: severe medical conditions (including known neurological, neurodegenerative or relevant somatic disorders, especially acute or chronic disorders affecting cognition and/or affectivity), the intake of centrally active medication, illegal drug use, and current pregnancy (cf. [25]). The following questionnaires were administered to all participants: the German version of the ASA-27 as well as the German versions of the Anxiety Sensitivity Inventory (ASI-3), the Spielberger State-Trait Anxiety Inventory—Trait subscale (STAI-T), the Beck Anxiety Inventory (BAI), the Agoraphobic Cognitions Questionnaire (ACQ), the Beck Depression Inventory (BDI-II), and the short form of the Center for Epidemiologic Studies Depression Scale (CES-D). The final sample size of 1520 individuals was based on the full availability of every item of the ASA-27. All participants provided written informed consent. The study was approved by the ethics committees of the universities of Würzburg, Münster, and Hamburg, Germany, and conducted in agreement with the Declaration of Helsinki in its latest version.

### Translation

In accordance with the guidelines outlined by the International Society of Pharmacoeconomics and Outcomes Research (ISPOR) in their guideline statement “Translation and Cultural Adaptation of Patient Reported Outcomes Measures—Principles of Good Practice” (PGP), a consecutive forward and backward translation was conducted. The original version of the ASA-27 [12] was translated independently into German from English by two investigators fluent in both languages. The translations were assessed by a team of psychiatrists, psychologists, and data scientists in a consensus meeting. The resulting first joint translated version (see supplementary information for the full questionnaire) was evaluated in a pilot group of participants, resulting in a revised German version, which was subsequently back-translated into English. Lastly, all authors involved in the translation process reached full consensus on a final German version of the ASA-27.

### Statistical analysis

Descriptive and frequency statistics were calculated for demographic variables and the ASA-27 total score. A multiple regression analysis was conducted to evaluate demographic variables as possible predictors of the overall total ASA-27 score. For reliability analysis, individual item and item-total statistics were calculated. Cronbach’s α was used as a measure of internal consistency and was checked for potential improvement by deleting individual items. To assess the factor structure of the German ASA-27, an unrotated principal component analysis was conducted (cf. [12]), and all factors with eigenvalues > 1.0 were extracted and analyzed for their factor loadings. Partial correlation analyses controlling for age and sex were performed to evaluate the concurrent validity of the German ASA-27 against the German versions of the ASI‑3, STAI‑T, BAI, and ACQ as well as the BDI-II and CES‑D. All statistical analyses were conducted in SPSS version 25. For all analyses, a significance level of *p* < 0.05 was applied.

## Results

### Descriptive statistics and ASA-27 score predictors

**Fig. 1:**
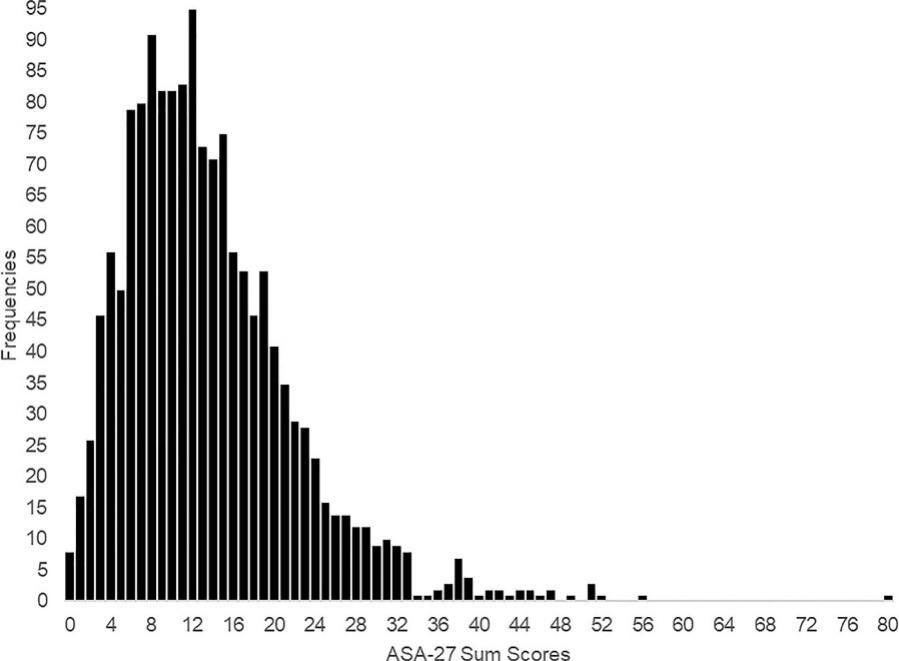
Distribution of Adult Separation Anxiety Questionnaire (*ASA-27*; German version) total sum scores (*n* = 1520)

The overall mean age of the sample was 25.0 years (*SD* = 5.8; female = 1090). The overall mean ASA-27 score was 13.6 (*SD* = 8.5; Fig. 1). For an overview on the demographic and psychometric properties of the study sample, see Table 1. Age and sex were both significant predictors of ASA-27 total scores (*F* = 33.2, *df* = 2, *p* < 0.001), with increased scores associated with younger age (β = −0.12, *t* = −4.65, *p* < 0.001) and female sex (β = −0.15, *t* = −5.88, *p* < 0.001).

**Table 1:** Demographic and psychometric characteristics of the study sample (*n* = 1520)

	Mean	*SD*
Age (years)	24.98	5.76
*Questionnaires*		
ASA-27	13.64	8.47
BDI-II	3.87	4.73
BAI	6.98	6.99
STAI‑T	33.54	7.91
ACQ	1.30	0.24
ASI‑3	12.70	8.80
*Sex*	**Total**	**Percentage**
Male	430	28.29%
Female	1090	71.71%

*SD* standard deviation, *ASA-27* Adult Separation Anxiety Questionnaire, *BDI-II* Beck Depression Inventory II, *BAI* Beck Anxiety Inventory, *STAI‑T* Spielberger State-Trait Anxiety Inventory—Trait subscale, *ACQ* Agoraphobic Cognitions Questionnaire, *ASI‑3* Anxiety Sensitivity Inventory 3

### Reliability analysis

For an overview of individual item and item–total statistics, see Table 2. The individual item mean was 0.50 (range: 0.07 [item 24] to 1.66 [item 1]) and the mean item–item correlation was 0.21 (range: 0.01 [items 1 and 26] to 0.51 [items 21 and 24]). Item–total correlation ranged from 0.21 (item 26) to 0.58 (item 18). A Cronbach’s α of 0.87 indicated good internal consistency for the complete translated ASA-27 scale, and deletion of individual items did not increase the overall alpha value (Cronbach’s α values ranged from 0.86 [e.g., item 18] to 0.87 [item 26]).

**Table 2:** Individual ASA-27 (German version) item and item–total statistics (*n* = 1520)

Item	Mean	SD	Item–total correlation	Cronbach’s *α* if item deleted
1. Feels more secure at home with close attachments	1.66	1.09	0.34	0.87
2. Experiences difficulty in staying away from home for several hours	0.08	0.31	0.27	0.87
3. Carries around something in purse or wallet for security or comfort	0.40	0.70	0.37	0.86
4. Experiences extreme stress when leaving home to go on a long trip	0.48	0.66	0.45	0.86
5. Suffers from nightmares or dreams about separation from close attachments	0.41	0.62	0.52	0.86
6. Experiences extreme stress before leaving someone close when going away on a trip	0.56	0.70	0.52	0.86
7. Becomes very upset when usual routine is disrupted	0.65	0.64	0.35	0.87
8. Worries about the intensity of relationships with close attachments	0.46	0.63	0.45	0.86
9. Experiences physical symptoms before leaving to go to work or other regular activities	0.23	0.47	0.37	0.86
10. Talks a lot in order to keep close attachments around	0.59	0.73	0.31	0.87
11. Concerned where close attachments are going when separated from them	0.23	0.48	0.45	0.86
12. Experiences difficulty in sleeping alone at night	0.79	0.91	0.51	0.86
13. Better able to sleep if he/she can hear the voices of close attachments or voices on the TV or radio	0.78	0.95	0.37	0.87
14. Becomes very distressed when thinking about being away from close attachments	1.20	0.78	0.57	0.86
15. Suffers from nightmares or dreams about separation from home	0.15	0.41	0.44	0.86
16. Worries about close attachments coming to serious harm	0.76	0.75	0.51	0.86
17. Becomes very upset with change to usual daily routine if it interferes with contact with close attachments	0.69	0.70	0.49	0.86
18. Worries about close attachments leaving	0.89	0.74	0.58	0.86
19. Sleeps better if the lights are on in the house or bedroom	0.12	0.41	0.30	0.87
20. Tries to avoid being at home alone when close attachments are out	0.22	0.54	0.48	0.86
21. Suffers from panic attacks when thinking about leaving close attachments or about them leaving	0.11	0.38	0.44	0.86
22. Anxiety about not speaking to close attachments on the telephone regularly	0.18	0.46	0.48	0.86
23. Afraid that he/she would not be able to cope if close attachments left	0.58	0.72	0.58	0.86
24. Suffers from panic attacks when separated from close attachments	0.07	0.29	0.43	0.86
25. Worries about possible events that may separate him/her from close attachments	0.43	0.63	0.46	0.86
26. Close attachments have mentioned that he/she talks a lot	0.67	0.85	0.21	0.87
27. Worries that relationships are so close it may cause others problems	0.26	0.52	0.38	0.86

*ASA-27* Adult Separation Anxiety Questionnaire, *SD* standard deviation

### Factor analysis

For an overview of factor loadings, see Table 3. Principal component analysis led to the extraction of six factors with eigenvalues > 1.0. Factor 1 explained 25.2% of the total ASA-27 variance, which was higher than the summed contribution of all subsequent factors (range: 6.4–4.0%), for a total explained variance of 49.7% across all factors. Factor 1 displayed positive factor loadings for all items (range: 0.24 [item 26] to 0.65 [item 23]) and included the highest loadings for all items except for items 2, 10, 19, 26, and 27.

**Table 3:** ASA-27 (German version) factor loadings from the unrotated principal component analysis (*n* = 1520)

Item	Factor					
	1	2	3	4	5	6
1. Feels more secure at home with close attachments	0.38	−0.30	−0.03	0.13	0.37	0.19
2. Experiences difficulty in staying away from home for several hours	0.32	0.05	0.05	−0.31	0.56	0.20
3. Carries around something in purse or wallet for security or comfort	0.41	−0.07	−0.04	0.06	0.17	−0.32
4. Experiences extreme stress when leaving home to go on a long trip	0.51	−0.05	−0.26	−0.19	0.37	−0.37
5. Suffers from nightmares or dreams about separation from close attachments	0.59	−0.14	−0.04	−0.03	−0.20	−0.38
6. Experiences extreme stress before leaving someone close when going away on a trip	0.59	−0.12	−0.25	−0.09	0.11	−0.39
7. Becomes very upset when usual routine is disrupted	0.41	0.27	−0.18	−0.25	0.14	0.22
8. Worries about the intensity of relationships with close attachments	0.51	0.40	−0.03	−0.04	−0.04	0.13
9. Experiences physical symptoms before leaving to go to work or other regular activities	0.43	0.12	0.13	−0.22	0.19	0.07
10. Talks a lot in order to keep close attachments around	0.33	0.50	0.17	0.47	0.21	−0.12
11. Concerned where close attachments are going when separated from them	0.52	0.09	−0.08	−0.13	0.08	0.24
12. Experiences difficulty in sleeping alone at night	0.55	−0.44	0.03	0.28	0.09	0.18
13. Better able to sleep if he/she can hear the voices of close attachments or voices on the TV or radio	0.42	−0.34	0.10	0.27	0.02	0.14
14. Becomes very distressed when thinking about being away from close attachments	0.62	−0.19	−0.32	0.22	−0.16	0.08
15. Suffers from nightmares or dreams about separation from home	0.51	−0.07	0.10	−0.19	−0.07	−0.28
16. Worries about close attachments coming to serious harm	0.57	−0.20	−0.24	0.03	−0.09	−0.02
17. Becomes very upset with change to usual daily routine if it interferes with contact with close attachments	0.56	0.22	−0.38	−0.04	−0.08	0.13
18. Worries about close attachments leaving	0.65	0.05	−0.13	0.11	−0.25	0.12
19. Sleeps better if the lights are on in the house or bedroom	0.35	−0.30	0.56	0.02	0.00	0.02
20. Tries to avoid being at home alone when close attachments are out	0.53	−0.32	0.29	0.06	0.16	0.12
21. Suffers from panic attacks when thinking about leaving close attachments or about them leaving	0.53	0.10	0.38	−0.23	−0.21	−0.18
22. Anxiety about not speaking to close attachments on the telephone regularly	0.56	−0.01	0.23	−0.07	−0.13	0.17
23. Afraid that he/she would not be able to cope if close attachments left	0.65	−0.02	−0.05	0.07	−0.31	0.07
24. Suffers from panic attacks when separated from close attachments	0.52	0.16	0.43	−0.31	−0.15	−0.03
25. Worries about possible events that may separate him/her from close attachments	0.53	0.14	−0.15	0.07	−0.14	0.12
26. Close attachments have mentioned that he/she talks a lot	0.24	0.43	0.21	0.59	0.19	−0.22
27. Worries that relationships are so close it may cause others problems	0.44	0.46	0.05	0.01	−0.02	0.12

*ASA-27* Adult Separation Anxiety Questionnaire

### Concurrent validity analysis

Partial correlation analyses revealed significant positive correlations of the ASA-27 score with the ASI‑3, the STAI‑T, the BAI, and the ACQ scores (*r* between 0.41 and 0.47, all *p* < 0.001). Significant correlations also arose between the ASA-27 and the BDI-II as well as the CES‑D (*r* = 0.43 and 0.38, respectively, both *p* < 0.001).

## Discussion

Separation anxiety disorder is a prevalent yet underdiagnosed anxiety disorder in adulthood. Hence, valid, internationally available psychometric instruments aiding the diagnostic process are urgently needed. In the present study, a validated German translation of the ASA-27 [12] is provided for the first time. The German translation of the ASA-27 demonstrates good internal consistency, comparable to those reported in previous studies [5, 6, 12, 18].

A principal component analysis revealed a factor structure consistent with other versions of the questionnaire [5, 6, 12, 18, 19]: A single primary factor explained the majority of the variance, displaying a consistent measurement of the unidimensional construct of SEPAD.

The concurrent validity of the German ASA-27 was demonstrated by significant correlations with other dimensional measures of anxiety and depressive symptoms. Analogous relationships of SEPAD with other anxiety components have been reported previously [9, 18, 19, 27, 28, 30]. This is not surprising, given the substantial overlap in comorbidity with other anxiety disorders (for reviews, see [2, 4, 11, 22]). However, these results also indicate the need for further research on the distinct and shared clinical features of SEPAD.

Consistent with epidemiological data [30], younger age and female sex were associated with higher ASA-27 scores, which reflects a well-established pattern across anxiety spectrum conditions [8, 13], and the negative correlation between age and ASA-27 scores is generally in line with the early age-of-onset of SEPAD.

By providing translated versions of the ASA-27, it is hoped to raise awareness of adult SEPAD and to improve the diagnostics of adult SEPAD, ultimately aiding in providing adequate treatment. So far, specific treatment protocols for SEPAD are scarce. Cognitive behavioral therapy is considered to be effective in treating SEPAD [3, 22]. Initial evidence has also been provided for psychodynamic treatment approaches [14], but clinical trials are lacking. Psychopharmacologically, antidepressant treatment with selective serotonin reuptake inhibitors (SSRI) or serotonin-norepinephrine reuptake inhibitors (SNRI) is recommended [22]. Furthermore, vilazodone, an SSRI and serotonin 1 A (5HT1A) receptor partial agonist, was found to be effective in reducing SEPAD symptoms in adults in a first clinical trial [26]. Instruments such as the ASA-27 could be applied routinely in a prevention context (cf. [7, 24]) or when addressing other mental disorders or psychological issues to screen for symptoms of SEPAD in light of its high comorbidity rates (cf. [11, 15–17, 22]). This is of particular relevance since aspects of SEPAD are often overlooked in standard clinical care, which may lead to impaired treatment response [1] and therefore contribute to the chronicity of anxiety disorders. In a research context, the assessment of SEPAD can also aid in identifying the neurobiological underpinnings of separation anxiety (cf. [20, 21]) as well as transdiagnostic aspects of emotional processing and anxiety patterns regarding mental health issues.

### Limitations

While this paper describes the first German-language instrument for the assessment of symptoms of SEPAD in adulthood, some limitations of the present validation study have to be considered. Firstly, no other SEPAD-specific measurement besides the ASA-27 was utilized to examine its concurrent validity. This is, however, complicated by the fact that no other instruments, neither self-report nor clinician-rated instruments, are available for the assessment of adult SEPAD in German. Furthermore, when interpreting the results, it has to be considered that only subjective self-ratings were collected, which are susceptible to various methodological problems, such as recall bias, answer tendencies, social desirability, or sequency effects. Eventually, the creation of a dedicated module within the Structured Clinical Interview for DSM‑5 (SCID-5-CV) would assist the adequate consideration of SEPAD in psychiatric and psychotherapeutic care. Moreover, the questionnaire was validated in a healthy population not affected by clinical levels of SEPAD or other mental disorders. Therefore, a subsequent validation in a patient population with SEPAD is desirable so as to address the specificity and sensitivity of the German ASA-27 translation. Validation in the context of a categorical SEPAD diagnosis would facilitate the verification of distinct cut-off values for SEPAD. While the original version of the ASA-27 has demonstrated favorable retest reliability [12], due to the present study’s design, it was not possible to evaluate the retest reliability of the German version. Future studies should thus employ a longitudinal design to assess the stability of ASA-27 scores over time.

## Practical conclusion

- The German ASA-27 emerged as a valid and reliable tool for assessing symptoms of separation anxiety in adults
- Thus, for the first time, a psychometric instrument is presented for the evaluation of SEPAD in German-speaking populations over the age of 18 as now incorporated in the taxonomy of the DSM‑5 and ICD-11.
- The provision of a high-quality translated version of the questionnaire is hoped to assist in the accurate assessment of SEPAD both in routine clinical care and in research environments.
- This will be helpful in facilitating cross-national and cross-cultural comparisons, but also day-to-day patient treatment.

## Supplementary Information

Deutsche Fassung des Adult Separation Anxiety Questionnaire (ASA-27) [German version of the Adult Separation Anxiety Questionnaire (ASA-27)])

## Data Availability

The data that support the findings of this study are available from the corresponding author upon reasonable request. The German version of the ASA-27 is available as part of the supplementary information.

## Declarations

All study procedures mentioned in this article were in accordance with the ethical standards indicated in each case.
