# Supplementary material for: The Adult Separation Anxiety Questionnaire (ASA-27): reliability of the German translation, factor structure, and concurrent validity with anxiety sensitivity and agoraphobic cognition
Source: Nervenarzt. 2025 Mar 17;96(7):678–85. doi: 10.1007/s00115-025-01806-w (PMC12662851; doi:10.1007/s00115-025-01806-w)
Supplement: Supplementary file 1 — Deutsche Fassung des Adult Separation Anxiety Questionnaire (ASA-27) [German version of the Adult Separation Anxiety Questionnaire (ASA-27)]) [file 115_2025_1806_MOESM1_ESM.pdf]

Deutsche Fassung des Adult Separation Anxiety Questionnaire (ASA-27)

ASA-27

Die folgenden Aussagen beziehen sich auf Symptome, die Sie möglicherweise im Erwachsenenalter (nach dem 18. Lebensjahr) hatten. Bitte kreuzen Sie zu jeder Frage die für Sie am besten zutreffende Antwort an, je nachdem, ob Sie diese Symptome bereits erlebt haben und wenn ja, in welcher Ausprägung. Bitte beantworten Sie jede Frage.

Das passiert mir…		sehr oft	ziemlich oft	gele-gentlich	nie
1	Haben Sie sich zuhause sicherer gefühlt, wenn Ihnen nahestehende Personen bei Ihnen waren?				
2	Haben Sie je Schwierigkeiten damit gehabt, für ein paar Stunden am Stück nicht zuhause zu sein				
3	Haben Sie etwas in Ihrer Handtasche oder in Ihrem Geldbeutel mit sich getragen, das Ihnen ein Gefühl von Sicherheit oder Trost/Wohlbefinden vermittelt?				
4	Haben Sie unter großer Anspannung gestanden, bevor Sie Ihr Zuhause verlassen haben, um eine längere Reise zu unternehmen?				
5	Haben Sie unter Albträumen oder Träumen gelitten, in denen Sie von einer nahestehenden Person getrennt wurden/waren?				
6	Standen Sie schon einmal unter großer Anspannung, bevor Sie eine nahestehende Person verlassen haben, um auf Reisen zu gehen?				
7	Haben Sie sich sehr geärgert, wenn Ihre gewohnten Tagesabläufe unterbrochen wurden?				
8	Waren Sie über die Intensität der Beziehung zu den Personen, die Ihnen am nächsten stehen, besorgt, z. B. dass Sie diesen zu eng verbunden sind?				
9	Haben Sie Symptome wie Kopfschmerzen, Bauchschmerzen, Übelkeit oder ähnliches verspürt, bevor Sie das Haus für die Arbeit oder irgendeine andere alltägliche Tätigkeit verlassen haben?				
10	Sind Sie der Meinung, dass Sie viel reden, um andere Menschen an sich zu binden?				
11	Waren Sie besonders besorgt darüber, wohin Ihnen nahestehende Personen gehen, wenn Sie von diesen Menschen getrennt sind, z. B. wenn Sie diese verlassen, um auf die Arbeit oder aus dem Haus zu gehen?				
12	Hatten Sie Schwierigkeiten damit, nachts alleine zu schlafen, bzw. schlafen Sie besser, wenn jemand, der Ihnen nahe steht, im Haus ist?				
13	Haben Sie bemerkt, dass Ihnen das Einschlafen leichter fällt, wenn Sie die Stimme von Ihnen nahestehenden Personen oder das Geräusch eines Fernsehers oder eines Radios hören?				
14	Hat es Sie betrübt, wenn Sie daran gedacht haben, von Ihnen nahestehenden Personen getrennt zu sein?				

15	Haben Sie unter Albträumen oder Träumen gelitten, in denen Sie weg von zuhause waren?				
16	Haben Sie sich viele Sorgen darüber gemacht, dass den Ihnen nahestehenden Personen etwas Schlimmes zustoßen könnte, zum Beispiel ein Autounfall oder eine schwere Erkrankung?				
17	Haben Sie sich sehr über Änderungen Ihrer Tagesabläufe geärgert, wenn diese den Kontakt mit Ihnen nahestehenden Personen störten?				
18	Haben Sie sich Sorgen darüber gemacht, dass Personen, die Ihnen wichtig sind, Sie verlassen könnten?				
19	Haben Sie bemerkt, dass Sie besser schlafen, wenn das Licht im Haus oder im Schlafzimmer an ist?				
20	Haben Sie versucht zu vermeiden, alleine zuhause zu sein, vor allem wenn Ihnen nahestehende Personen nicht da sind?				
21	Haben Sie unter plötzlichen Anfällen von Angst oder Panikattacken (z. B. plötzliches Zittern, Schwitzen, Kurzatmigkeit, Herzklopfen) gelitten, wenn Sie daran gedacht haben, Ihnen nahestehende Personen zu verlassen oder dass diese Sie verlassen?				
22	Haben Sie bemerkt, dass Sie Angst bekommen, wenn Sie nicht regelmäßig (z. B. täglich) mit Ihnen nahestehenden Personen telefonieren?				
23	Hatten Sie Angst, dass Sie es nicht bewältigen könnten oder nicht weitermachen könnten, wenn Sie jemand, der Ihnen wichtig ist, verlassen würde?				
24	Haben Sie unter plötzlichen Anfällen von Angst oder Panikattacken (z. B. plötzliches Zittern, Schwitzen, Kurzatmigkeit, Herzklopfen) gelitten, wenn Sie von Ihnen nahestehenden Personen getrennt waren?				
25	Haben Sie sich viele Sorgen über mögliche Ereignisse gemacht, die Sie von den Ihnen nahestehenden Personen trennen könnten, z. B. aufgrund von Arbeitsbedingungen/-anforderungen?				
26	Haben Ihnen nahestehende Menschen schon einmal erwähnt, dass Sie viel reden?				
27	Haben Sie sich Sorgen darüber gemacht, dass Ihre Beziehungen zu einigen Menschen so eng sind, dass das für diese Menschen problematisch werden könnte?				

Seuling, P. D., Gottschalk, M. G., Vietz, M., Lueken, U., Lonsdorf, T. B., Dannlowski, U., Pauli, P., Deckert, J., Pini, S., Manicavasagar, V., Domschke, K., Schiele, M. A. The Adult Separation Anxiety Questionnaire (ASA-27): Reliability of the German Translation, Factor Structure and Concurrent Validity with Anxiety Sensitivity and Agoraphobic Cognition. *Nervenarzt,* 2025. https://doi.org/10.1007/s00115-025-01806-w

Kontakt: PD Dr. M. A. Schiele
Universitätsklinikum Freiburg
Klinik für Psychiatrie und Psychotherapie
Hauptstraße 5, 79104 Freiburg
E-Mail: miriam.schiele@uniklinik-freiburg.de
